# Force Estimation during Cell Migration Using Mathematical Modelling

**DOI:** 10.3390/jimaging8070199

**Published:** 2022-07-15

**Authors:** Fengwei Yang, Chandrasekhar Venkataraman, Sai Gu, Vanessa Styles, Anotida Madzvamuse

**Affiliations:** 1 Department of Engineering, University of Warwick, Coventry CV4 7AL, UK; fengwei.yang@warwick.ac.uk (F.Y.); sai.gu@warwick.ac.uk (S.G.); 2 Department of Mathematics, School of Mathematical and Physical Sciences, University of Sussex, Brighton BN1 9QH, UK; c.venkataraman@sussex.ac.uk (C.V.); v.styles@sussex.ac.uk (V.S.); 3 Department of Mathematics, University of British Columbia, 1984 Mathematics Rd., Vancouver, BC V6T 1Z2, Canada

**Keywords:** cell migration, optimal control, geometric partial differential equations, mechanical membrane forces, cell polarisation

## Abstract

Cell migration is essential for physiological, pathological and biomedical processes such as, in embryogenesis, wound healing, immune response, cancer metastasis, tumour invasion and inflammation. In light of this, quantifying mechanical properties during the process of cell migration is of great interest in experimental sciences, yet few theoretical approaches in this direction have been studied. In this work, we propose a theoretical and computational approach based on the optimal control of geometric partial differential equations to estimate cell membrane forces associated with cell polarisation during migration. Specifically, cell membrane forces are inferred or estimated by fitting a mathematical model to a sequence of images, allowing us to capture dynamics of the cell migration. Our approach offers a robust and accurate framework to compute geometric mechanical membrane forces associated with cell polarisation during migration and also yields geometric information of independent interest, we illustrate one such example that involves quantifying cell proliferation levels which are associated with cell division, cell fusion or cell death.

## 1. Introduction

Cell migration is a fundamental cellular process that is essential to life and is linked to many important physiological and pathological events such as the immune response, wound healing, tissue differentiation, embryogenesis, and tumour invasion [1,2,3,4,5,6,7].

During migration, mechanical processes play a pivotal role, for example cellular biomechanics direct its physical behaviour, as well as its cellular functions in the biological context of health and disease [8,9,10]. Cells also physically interact with their extracellular environments via mechanical forces, for example, cell division, apoptosis, bleb and mitosis [11,12,13]. The strength of the forces varies as the sensitivity of a cell evolves with surrounding biomechanical and biochemical stimulus [11].

A key determinant of cellular biomechanics is the actin cytoskeleton [8]. It contains dynamic actin architectures that continuously re-arrange and turnover. The cytoskeletal forces are exerted on a plasma membrane, which define and insure the stability of the interior of the cell [14]. At the leading edge, a protrusion force is generated by actin architectures. Membrane tension balances these locally imposed forces and ensures rear retraction [10]. The characteristic time scale is short, often sub-seconds. The measured forces suggest they may range from Pico-Newtons (pN) to Micro-Newtons (μN) [10,14]. The cellular force generation intertwines with many other processes, forming a complex system. In addition, a noticeable change in a single cell behaviour may lead to a significant event on a tissue scale, so unravelling the mutual interplay between physical interactions such as protrusion and retraction forces are essential to understanding cell dynamics [10].

According to the research by Lieber et al., in 2013 [10] and Barbieri et al. in 2021 [8], there is very little understanding and limited ways to quantify cellular forces. The former claims a hypothesis on how membrane tension is set and regulated by cells, but states there is very little evidence to either support or disprove it; the latter describes challenges in quantification due to technical constraints.

Traditionally, our understanding of cell dynamics often comes from visual inspection using high-throughput, high-resolution microscopy and related imaging techniques [8,15]. Phase-contrast microscopes utilise partially coherent illumination to extract quantitative phase data [16,17]. Interferometric-based techniques make use of the Fourier decomposition [18,19]. Other alternative techniques include optical coherence tomography [20] and digital holographic microscopy [15,21]. Additionally to imaging, Simson et al. in [9] reports an interferometric technique to measure bending modulus, membrane tension and adhesion energy. A mechanic-optical biosensor is described in [22] to sense local cell adhesive forces. In [10] the authors discuss about membrane fluctuations and [23] summarises soft polymers that are typically used to measure cellular forces.

In this study, we propose an alternative theoretical and computational approach whereby, instead of measuring physical quantities in experiments, we describe the underlying rules (and often hypotheses) using mathematical equations, thereby obtaining a model for a migrating cell which incorporates certain assumptions on the physics underlying migration. There have been a number of studies carried out using simulations of such models to model cell migration, e.g., in [24] and subsequent related works, a phase-field model for keratocyte migrations is developed, in [25], some quantitative predictions are derived on how adhesion geometry and stiffness change cell behaviour. In this paper, we approach the problem of membrane force estimation during cell migration as the problem of computing forces such that we fit an established model of cell migration [26,27] to microscopy data that provides the cell membrane position at a series of time points. Specifically, we use the frames of imaging data to extract the position of the cell membrane at a series of times and use this data in an optimal control model as our target positions for the position of the cells under our mathematical model at the corresponding times. The control which is computed to minimise the difference between the cell positions generated computationally and the data corresponds to the protrusive force active at the cell membrane. This approach, i.e., the optimal control of phase field models albeit in a different context has received recent interest e.g., [28]. Computational simulations also help to build devices which can then be used to directly measure cellular properties, such as the microsystems summarised in [11].

The major novelties of the present work with respect to [26,27] are threefold: firstly, an application of the optimal control approach to time series data from real experiments, secondly, parameterisation of the model allowing the control problem to be interpreted as an approach for the estimation of forces during cell migration and finally, the application of the approach to the biologically important problem of quantifying cell division (or apoptosis) rates in a population of cells. These novelties, transform the approach from something that is of mainly theoretical interest to something that is of considerably utility to biological practitioners.

This paper is organised as follows. In Section 2, we describe our theoretical and computational modelling approach. We take experimental observations of cell migration from three different cell types: keratocyte in [29], epithelial bladder cancer cell from the T24 cell line in [30], and epithelial kidney cancer cell from the MDCK cell line in [31]. Using our theoretical model, we re-create the corresponding computational cells and compute the predicted membrane forces. In Section 3, we present our results. We conclude our results in Section 5.

## 2. Materials and Methods

### 2.1. Mathematical Model for Membrane Force Estimation

We consider a volume-constrained Allen-Cahn equation with forcing as the “forward model” in an optimal control approach to whole cell tracking. The model arises from considering a simplified force balance on the cell membrane, further details on the model, the corresponding sharp interface formulation and its physical justification are provided in [26]. The volume constrained Allen-Cahn equation with forcing, a diffuse interface approximation to forced mean curvature flow, is stated as follows

(1) $$\left\{\begin{array}{cc} \epsilon \tau \partial_{t}\phi \left(\vec{x},t\right)=\delta \epsilon ▵\phi \left(\vec{x},t\right)-\frac{\delta }{\epsilon }G^{\prime }\left(\phi \left(\vec{x},t\right)\right)+\eta \left(\vec{x},t\right)+\lambda \left(t\right),\; & \mathrm{in}\;\Omega \times (0,T],\\ \phi \left(\vec{x},0\right)=\phi^{0}\left(\vec{x}\right),\; & \mathrm{in}\;\Omega ,\\ \nabla \phi \left(\vec{x},t\right)\cdot \nu_{\Omega }\left(\vec{x}\right)=0,\; & \mathrm{on}\;\partial \Omega . \end{array}\right.$$

In the above model, $\phi (\vec{x},t)$ is a phase field variable whose zero level set corresponds to the cell membrane; ϵ, satisfying 0<ϵ≪1 is a parameter governing the interfacial width of the diffuse interface, $G\left(\phi \right)=\frac{1}{4}{\left(1-\phi^{2}\right)}^{2}$ is a double well potential which has minima at ±1 and λ is a time-dependent constraint on the mass of the cell that models a volume constraint [32]. In practice our constraint differs from conservation of mass since the target data may have differing ‘mass’ (effectively cell area) from image to image. The initial condition $\phi^{0}\left(\vec{x}\right)$ is taken as the initial image from the experimental observations. $\nu_{\Omega }$ is the outward normal to ∂Ω.

Without loss of generality we assume the domain $\Omega ={\left[0,L\right]}^{2}$. Biologically, $\phi (\vec{x},t)$ can be viewed as a volume fraction (ϕ≈1 in the cell bulk, ϕ≈−1 in the extracellular matrix, ϕ≈0 on the cell membrane), ϵ as the thickness of cell membrane, τ is an effective friction due to the interaction with the extracellular matrix, δ is the surface tension and since we focus on the two-dimensional, rather than three-dimensional cases, we average assuming a constant cell height of 0.1 μm.

In our modelling framework, $\eta (\vec{x},t)$ is a membrane force generated by the cell during migration. A positive η indicates a protrusive force that drives the cell forward, while a negative force corresponds to a retractive force that allows the cell to contract enabling the cell to dislocate from the substrate to move its body forward. We make the assumption that the cells move as a result of forces that are only exerted in a region close to the membrane which is biologically reasonable since forces leading to migration are primarily exerted in the the actin cortex which is a thin region close to the membrane [26,27].

The physical interpretation of the model variables in Equation (1) is given in Table 1 below.

Next we give the physical units of the variables in Model (1), their balance can be interpreted as follows

(2) $$\begin{array}{cc} \; & \underset{\mu m}{\underset{︸}{\epsilon }}\;\underset{pNs/\mu m^{2}}{\underset{︸}{\tau }}\;\underset{1/s}{\underset{︸}{\partial_{t}\phi }}\;\\ \; & =\;\underset{pN}{\underset{︸}{\delta }}\;\underset{\mu m}{\underset{︸}{\epsilon }}\;\underset{1/\mu m^{2}}{\underset{︸}{▵\phi }}\;+\;\frac{\overset{\overset{pN}{︷}}{\delta }}{\underset{\underset{\mu m}{︸}}{\epsilon }}\;G^{\prime }\left(\phi \right)\;+\;\underset{pN/\mu m}{\underset{︸}{\eta }}+\;\underset{pN/\mu m}{\underset{︸}{\lambda }}. \end{array}$$

In the above equation, the units are μm:micrometer, s:second, and pN:pico−newton. In Table 2 we present the values we use for the parameters in the model, together with the references that gave rise to these choices.

From here onwards, we wish to work with a unit-free model, and to this end, we introduce the dimensionless variables

(3) $$\bar{\vec{x}}=\frac{\vec{x}}{L},\;\bar{\epsilon }=\frac{\epsilon }{L},\;\bar{t}=\frac{t}{T},\;\bar{\eta }=\frac{\eta }{F/L},\;\bar{\delta }=\frac{\delta }{F},\;\bar{\tau }=\frac{\tau }{FT/L^{2}},$$

where the characteristic scale *F* is related to the kinetic scaling.

Applying the above scaling, we obtain the following dimensionless model

(4) $$\left\{\begin{array}{cc} \epsilon \bar{\tau }\frac{\partial \phi (\bar{\vec{x}},\bar{t})}{\partial \bar{t}}=\bar{\delta }\epsilon ▵\phi \left(\bar{\vec{x}},\bar{t}\right)+\frac{\bar{\delta }}{\bar{\epsilon }}G^{\prime }\left(\phi \left(\bar{\vec{x}},\bar{t}\right)\right)+\bar{\eta }\left(\bar{\vec{x}},\bar{t}\right)+\lambda \left(\bar{t}\right),\; & \mathrm{in}\;\bar{\Omega }\times \bar{I},\\ \phi \left(\bar{\vec{x}},0\right)=\phi^{0}\left(\bar{\vec{x}}\right),\; & \mathrm{in}\;\bar{\Omega },\\ \nabla \phi \left(\bar{\vec{x}},\bar{t}\right)\cdot \nu_{\bar{\Omega }}\left(\bar{\vec{x}}\right)=0,\; & \mathrm{on}\;\partial \bar{\Omega }, \end{array}\right.$$

where $\bar{\Omega }={\left[0,1\right]}^{2}$ and $\bar{I}=\left(0,1\right]$.

### 2.2. Formulation and Approximation of the Optimal Control Problem and Biological Interpretation

The approach we consider can be thought of as finding a forcing term $\bar{\eta }\left(\bar{\vec{x}},\bar{t}\right)$ in Equation (4) such that the model best-fits the images. The methodology was first proposed in [26]; it sought to find $\bar{\eta }\left(\bar{\vec{x}},\bar{t}\right)$ that minimises the following objective functional

$$J\left(\eta ;\phi \right)=\frac{\theta }{2}\int_{0}^{T}\int_{\Omega }\eta^{2}\left(\bar{\vec{x}},t\right)d\bar{\vec{x}}dt+\frac{1}{2}\sum_{i=1}^{N_{obs}}\int_{\Omega }{\left(\phi \left(\bar{\vec{x}},t_{i}\right)-\phi_{obs,i}\left(\bar{\vec{x}}\right)\right)}^{2}d\bar{\vec{x}},$$

where $N_{obs}$ is the number of images we wish to fit to, $\phi_{obs,i}\left(\bar{\vec{x}}\right)$ is a (phase-field) representation of the data we wish to fit to at time $t_{i}$ and θ>0 is a regularisation parameter that is necessary for the optimal control problem to be well posed. We omit the details on the formulation and solution of the optimal control problem in this primarily applications focussed work, referring the interested reader to references [26,27].

The primary computational work in solving the optimal control problem lies in approximating the solution to Equation (1) which (typically) must be carried out a number of times. We refer the reader to [34,35,36] and references within for more details on phase-field models and their solution methods. We previously developed two approaches for the approximation of the optimal control problem, a finite element approach in [26] and an adaptive, parallelised finite difference approach in [27], which is based on geometric multi-grid methods. The computational cost in fitting to the multiple datasets that we consider below prompts us to use the more efficient approach of [27] in this work and we refer to [27] for further details on the space-time discretisation. For completeness, we briefly describe our approach for obtaining the results presented in this paper in the Appendix A.

We note that the computed $\bar{\eta }\left(\bar{\vec{x}},\bar{t}\right)$ can be interpreted physically as the membrane force (protrusive or retractive) required such that the motion of the boundary of the simulated cells under the model best resembles that of the imaging data. The interpretation of this $\bar{\eta }\left(\bar{\vec{x}},\bar{t}\right)$ we employ in the present work is that it corresponds to the estimate of the forces exerted on the membrane, e.g., F-Actin based protrusion or myosin based contraction which govern the motion of the cell.

### 2.3. Model Parameters for the Different Biological Datasets

In Table 3 we give the spatial and temporal settings associated with the three cell types to which we apply our model: keratocyte in [29], epithelial bladder cancer cell from the T24 cell line in [30], and epithelial kidney cancer cell from the MDCK cell line in [31].

## 3. Results

**Figure 1:**
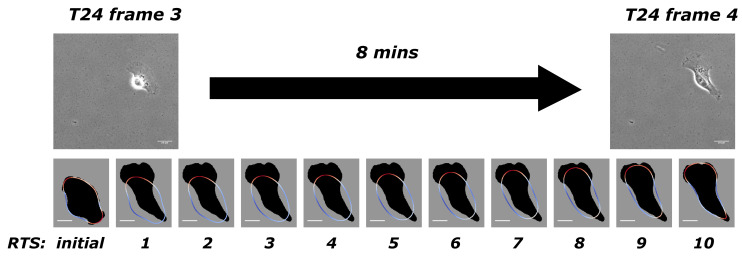
The first row illustrates two adjacent frames from the T24 experimental data [30] that were taken 8 min apart. The second row shows the initial shape and computed solutions at 10 intermediate time steps accordingly. The dark shadow in the background shows the targeted shape as the objective, which is the shape of the cell from frame 4. Bars indicate 20 μm.

For each of the biological datasets considered, we treat the initial frame of the video as data used to generate the initial conditions for the model. The remaining frames in the dataset are the target data we seek to fit the model to. Further details on our approach to extracting a phase field representation of the cell from imaging data are given in [26,27]. Our approach gives us the computed cell positions together with the estimated force such that the motion of the cells under our model recreates the observed motion from the imaging data. We use Figure 1 to illustrate our model when applied to experimental data from T24 cell line [30]. There is an 8-min gap between two adjacent frames in this experiment, and we take frames 3 and 4, for example. Our discretisation yields 10 time steps between these two frames. The first row in Figure 1 has two adjacent frames from the experimental data, and the second row shows the initial computed cell outline (obtained from the previous computation covering frames 2 to 3) and solutions at the 10 time steps with the computed optimal force. The solution from the 10th time step would then be used as the initial shape to compute the next stretch between frames 4 and 5. The dark shadow in the background shows the target shape as the objective, which is the shape of the cell from frame 4. This process continues successively throughout the full dataset.

**Figure 2:**
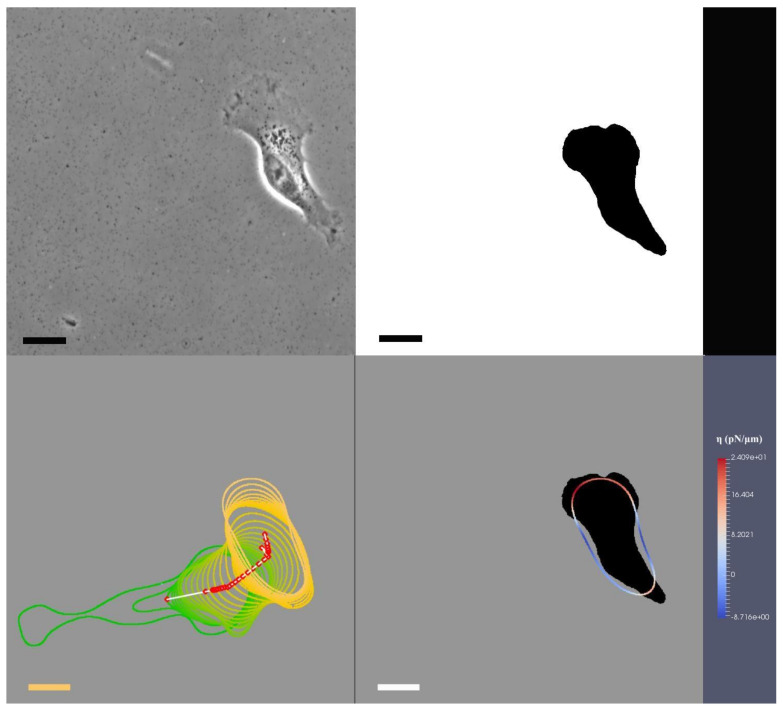
(**Top-left**): The original image from experimental observation; (**top-right**), The segmentation of the T24 cancer cell from the image; (**bottom-left**): We define the interfacial region of the cell and its centroid position. Within this sub-figure, we continually overlay the cell shapes and positions as the cell migrates; (**bottom-right**): We use colour coding to identify red as protrusion and blue as retraction forces and the locations they are exerted on the cellular interfacial region. Bars indicate 20 μm.

As an example in Figure 2, we present the first frame of our results on T24. We show the original image from T24 experiment [30] on the top-left; our segmentation of the shape of the T24 cancer cell on the top-right (this segmentation technique is a combination of Otsu and edge detection, we refer the reader to [27,37] for more details); on the bottom-left, we demonstrate the interfacial region of the cell, its centroid position, and we continually overlay the cell shapes as the cell migrates. On the bottom right, we show the exerted forces where we use colour coding (red as protrusion and blue as retraction) to illustrate the location and amount of forces exerted on the cellular interfacial region, representing the cell membrane

In Table 4, we summarise both protrusion and retraction forces re-created during the simulations of keratocyte migration (shown in Figure 2 and video in Appendix A). Each experimental image serves as a starting position or a goal. Our estimated forces are evaluated between adjacent frames. For the keratocyte [29], the actual real-world time between frames is 20 s. In Table 4, we show the average cell membrane length from our simulation in μm, the accumulated forces, the number of reconstructed time steps (denoted by RTS throughout), and the percentage of cell membrane where protrusion or retraction forces are exerted.

Our results on the epithelial bladder cancer cell T24 [30] are shown in Table 5. The layout of the table and its corresponding video in Appendix A are very similar to the keratocyte simulation.

The results of the epithelial kidney cancer cell MDCK [31] are shown in Table 6 and are presented in the similar manner, apart from an additional diagram shown in the corresponding video in Appendix A on the right-hand side.

We note that making direct comparisons of our results with experimental studies is challenging since other forces relevant to migration, such as traction forces generated through interactions with the substrate, are neglected in our model and often membrane forces are not directly measured. However, our results are consistent with available experimental results which attempt to measure retractive and/or protrusive forces generated by migrating cells; these results conclude that the total force exerted by the cells is of the order of 10s of Nano-Newtons [38].

### Geometric Quantities That Are of Biological Interest

Based on the computed phase field information, we can compute important and biologically relevant geometric information that is of significant interest to experimentalists. Through simple post-processing of the model outputs, we may obtain geometric quantities such as circularity, curvature and elastic energy of the membrane. We demonstrate one such post-processed quantity of interest by considering a dataset that consists of multiple cells undergoing division and demonstrating how our approach allows us to quantify cell proliferation rates, and also demonstrate geometrically, the cell division process. To proceed, we first state how to compute the Euler number of the cell membranes which in effect corresponds to the total number of cells present in the simulation. The Euler number, in two dimensions in the phase-field formulation is given by [39]

(5) $$N_{\epsilon }=\frac{1}{2\pi c}\int_{\left\{|\phi_{\epsilon }|<c\right\}}\left(-\Delta \phi_{\epsilon }+\frac{\nabla |\nabla \phi_{\epsilon }{|}^{2}\cdot \nabla \phi_{\epsilon }}{2|\nabla \phi_{\epsilon }{|}^{2}}\right)d\bar{\vec{x}}.$$

Here $\phi_{\epsilon }$ is the computed phase-field function. As this number corresponds to the number of cells present, it is extremely useful as it gives us the means to automatically track events such as cell division and cell fusion during the process of cell migration. This approach is extremely valuable as it could automate an otherwise laborious task of counting division, fusion or death events and removes the need for genetic manipulations which would be required to highlight such events. We illustrate this in the video in Appendix A related to the kidney cancer cell MDCK [31] where a number of cell divisions occur during the video and these are tracked accurately by the Euler number of the computed phase field.

**Figure 3:**
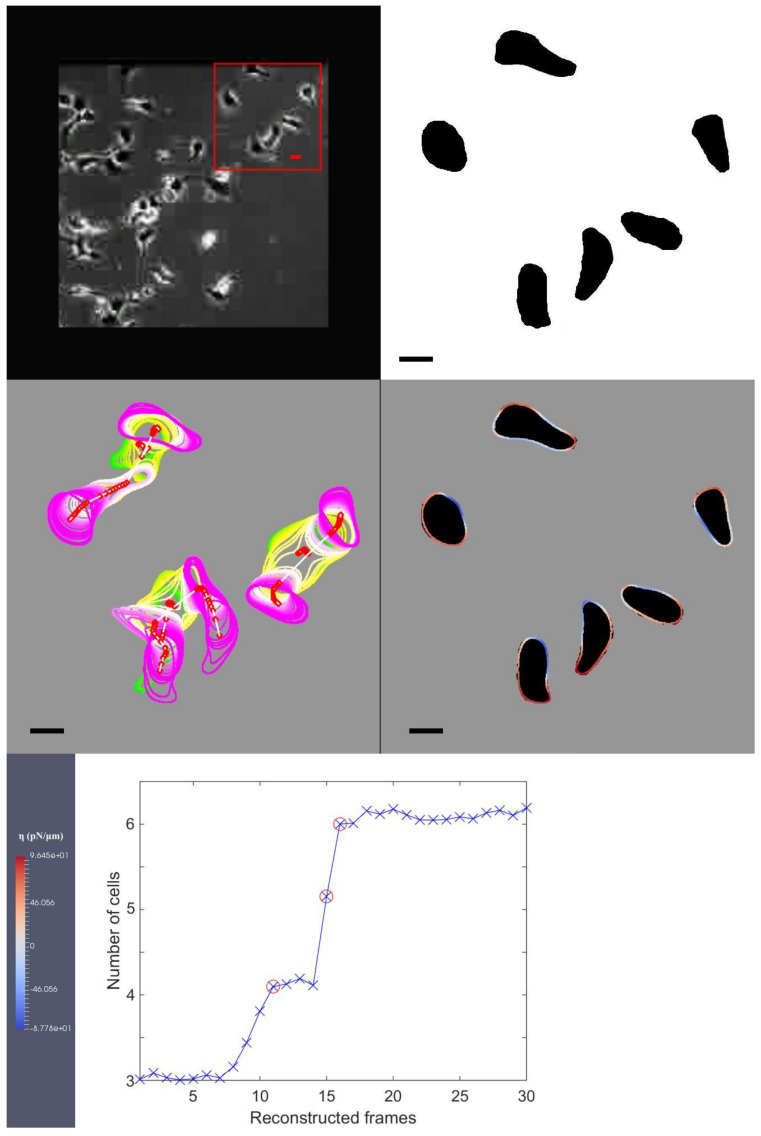
(**The first image on the first row**): The original image from experimental observation and the choice of the initial three cells which are used in the simulation. In this figure, it is the last frame of the data. **The second image on the first row**: The segmentation of the cells from the image on the left. (**The first image on the second row**): We define the interfacial region of the cell and its centroid position, within this sub-figure, we continually overlay the cell shapes and their positions as they migrate. (**The second image on the second row**): We use colour coding to identify red as protrusion, and blue as retraction forces and the locations they are exerted on the cellular interfacial region, the dark shadows in the background illustrate the targeted shapes that model (1) replicates. The bar on the right-hand side shows the maximum and minimum amount of forcing that the colour coding is illustrating. (**The only image on the third row**): We show the Euler number from Equation (5) computed at each RTS and red circles indicate the events of cell division. Bars indicate 20 μm.

For completeness, we include the final frame of the result video on the kidney cancer cell MDCK in Figure 3. In this figure, the original image from the experimental observation is shown as the first image on the first row, with a red box highlighting our choice of three cells used in our simulation. The second image on the first row illustrates the segmentation of the corresponding cells in the first image on the first row. The first image on the second row shows the interfacial region of the selected cells and their centroid points. In this sub-figure, we continually overlay the cell shapes and portions as they migrate. We use red for protrusion and blue for retraction forces to identify the regions where they are exerted around the cellular interfacial region. The dark shadows in the background illustrate the targeted shapes our model is replicating, and the bar on the right-hand side shows the maximum and minimum amount of forcing that the colour coding is illustrating. The only image on the third row shows our Euler number from Equation (5) computed at each RTS. Within this data, the initial three cells are divided into six cells, and we use red circles to indicate the cell division events in this graph.

## 4. Discussion

Single or population cell migration is essential for many biological processes such as immune response, embryogenesis, gastrulation, wound repair, cancer metastasis, tumour invasion, inflammation and tissue homeostasis. However, aberrant or defects in cell migration lead to various abnormalities and life-threatening medical conditions [40,41,42]. Increasing our knowledge on cell migration can help abate the spread of highly malignant cancer cells, reduce the invasion of white cells in the inflammatory process, enhance the healing of wounds and reduce congenital defects in brain development that lead to mental disorders. While single-cell sequencing has accelerated breakthroughs in cancer research and transformed our understanding of tumour biology, leading to significant impacts for cancer treatments, understanding and quantifying membrane force generation associated with cell migration remains an open problem, in this study we have exploited our modelling approach of using optimal control of surface geometric partial differential equations posed on the cell membrane to predict and estimate biological quantities of interest, such as protrusion and retraction forces. Our approach is substantially different from current-state-of-the-art modelling of such forces, it sets premises to study cell migration through complex multi-dimensional environments where force generation between the cell and the extracellular matrix is critical [40,41,42].

## 5. Conclusions

A number of recent studies such as [10] remark that small changes in mechanical forces generated from individual cells can lead to fundamental changes at tissue levels. However, as [8] indicates, it is technologically challenging to simply measure those forces during experiments, such as during the process of cell migration.

In this work, we estimate the forces exerted by migrating cells by computing ‘optimal’ forces such that a mathematical model for cell migration best fits observed imaging data. In this paper, we took experimental data of three different cell types: keratocyte in [29], epithelial bladder cancer cell from the T24 cell line in [30], and epithelial kidney cancer cell from the MDCK cell line in [31]. For each case, we demonstrate how we re-create the observed cell migration and summarise the protrusion and retraction forces generated under our model. We also note our approach is applicable to multiple cells and can be applied in three dimensions, given appropriate datasets in 3D. Our approach could also allow us to access biologically relevant quantities such as membrane length, circularity and curvatures. We demonstrate one example using the MDCK cell line dataset [31] in which a number of cell divisions occur during the evolution. Our approach deals robustly with this setting allowing accurate quantification of cell proliferation rates which is generally cumbersome if carried out manually. Moreover, we provide a means of tracking (automatically) the number of cells present which could be of practical use if one wishes to measure the rate of cell divisions, cell death or cell fusion.

Our proposed approach is amenable to further improvements and these include computing more accurate measurements of parameters such as friction force and surface tension as well as more refined modelling of migration itself. We note that it would be relatively straightforward to extend our simulations to three space dimensions, which would enable the recreation of more accurate cells and their environments [27] but a major challenge in this case is obtaining sufficiently high-resolution imaging data. As [43] states, cell-matrix adhesions and cytoskeletal organisation could be different in 2D and 3D measurements, and may alter key cell responses, including morphology, migration and proliferation. We also note that in principle this approach can be adapted to more complex models of cell migration and this is merely a proof-of-concept study illustrating the utility of our approach.

## Figures and Tables

**Table 1:** Physical interpretation of the model variables in Equation (1).

Parameter	Description
ϵ	Membrane thickness
τ	Friction
ϕ	Volume fraction
$\vec{x}$	Spatial coordinate
*t*	Time variable
*L*	Domain length
*T*	Time of entire experiment
δ	Surface tension
η	Forcing exerted on cell membrane

**Table 2:** Values of physical parameters used in Model (1).

Parameter	Description	Value
δ	Surface tension	1–10 pN [14,24,33]
τ	Friction force	≈2.62 pNs/µm${}^{2}$ [24]
ϵ	Membrane thickness	≈1.0 μm [24]
η	Forcing exerted on cell membrane	Fitting variable to be computed
		in the remainder of this paper

**Table 3:** Model parameters for the three different biological datasets.

Parameter	Description	Value
keratocyte		
*x*	Domain length in *x* axis	81.5μm
*y*	Domain length in *y* axis	81.5μm
*t*	Length of time	360 s
$T_{I}$	Time interval between frames in the video	20 s
*L*	Characteristic length	81.5μm
Υ	Characteristic time	360 s
*F*	Characteristic force for surface tension	10 pN
$\bar{\tau }$	(Dimensionless) friction force	4.85
$\bar{\epsilon }$	Numerical interfacial width	0.01
RTS	No. Reconstructed Time Steps between frames	10
T24		
*x*	Domain length in *x* axis	170 μm
*y*	Domain length in *y* axis	170 μm
*t*	Length of time	1920 s
$T_{I}$	Time interval between frames in the video	480 s
*L*	Characteristic length	170 μm
Υ	Characteristic time	1920 s
*F*	Characteristic force for surface tension	10 pN
$\bar{\tau }$	(Dimensionless) friction force	3.16
$\bar{\epsilon }$	Numerical interfacial width	0.005
RTS	No. Reconstructed Time Steps between frames	10
MDCK		
*x*	Domain length in *x* axis	220 μm
*y*	Domain length in *y* axis	220 μm
*t*	Length of time	1800 s
$T_{I}$	Time interval between frames in the video	300 s
*L*	Characteristic length	220 μm
Υ	Characteristic time	1800 s
*F*	Characteristic force for surface tension	10 pN
$\bar{\tau }$	(Dimensionless) friction force	7.04
$\bar{\epsilon }$	Numerical interfacial width	0.05
RTS	No. Reconstructed Time Steps between frames	5

**Table 4:** Details of estimating the membrane forces and its evolution through cell morphology reconstruction of the keratocyte. C. M. is an abbreviation for cell membrane. We note here that the Lagrange multiplier λ(t) in (1) is in effect a global spatially constant volume constraint force which is not included in the totals stated above.

Time Duration.	Avg. C. M.	Total Protrusion	Over % of	Total Retraction	Over % of
between Frames	Len. (µm)	Force (pN)	C. M.	Force (pN)	C. M.
1–2 (20 s)	94	33,480	75.3	5637	24.7
2–3 (20 s)	97	34,457	70.0	8253	30.0
3–4 (20 s)	97	37,047	73.8	7999	26.2
4–5 (20 s)	98	36,450	70.0	9301	30.0
5–6 (20 s)	98	37,076	70.3	10,334	29.7
6–7 (20 s)	99	36,338	70.2	10,688	29.8
7–8 (20 s)	100	36,076	70.5	10,722	29.5
8–9 (20 s)	101	38,377	71.6	9625	28.4
9–10 (20 s)	101	38,553	68.9	10,630	31.1
10–11 (20 s)	102	38,768	72.2	9723	27.8
11–12 (20 s)	88	37,751	68.2	10,788	31.8
12–13 (20 s)	90	36,796	70.4	10,001	29.6
13–14 (20 s)	89	40,149	72.4	9289	27.6
14–15 (20 s)	90	38,893	68.6	10,743	31.4
15–16 (20 s)	91	39,012	69.0	11,296	31.0
16–17 (20 s)	92	42,167	71.1	11,013	28.9
17–18 (20 s)	92	39,910	69.7	10,920	30.3
18–19 (20 s)	93	39,074	67.8	11,753	32.2

**Table 5:** Details of estimating the membrane forces and its evolution through cell morphology reconstruction of the T24. C. M. is an abbreviation for cell membrane. We note here that the Lagrange multiplier λ(t) in (1) is in effect a global spatially constant volume constraint force which is not included in the totals stated above.

Time Duration.	Avg. C. M.	Total Protrusion	Over % of	Total Retraction	Over % of
between Frames	Len. (µm)	Force (pN)	C. M.	Force (pN)	C. M.
1–2 (8 min)	224	165,361	43.8	134,424	56.2
2–3 (8 min)	178	212,371	55.6	48,428	44.4
3–4 (8 min)	191	161,132	57.6	37,831	42.4
4–5 (8 min)	175	173,554	51.0	45,541	49.0

**Table 6:** Details of estimating the membrane forces and its evolution through cell morphology reconstruction of the MDCK. C. M. is an abbreviation for cell membrane. We note here that the Lagrange multiplier λ(t) in (1) is in effect a global spatially constant volume constraint force which is not included in the totals stated above.

Time Duration.	Avg. C. M.	Total Protrusion	Over % of	Total Retraction	Over % of
between Frames	Len. (µm)	Force (pN)	C. M.	Force (pN)	C. M.
1–2 (5 min)	427	256,865	48.7	186,380	51.3
2–3 (5 min)	730	617,225	69.0	183,354	31.0
3–4 (5 min)	1063	692,221	61.9	466,208	38.1
4–5 (5 min)	1066	566,266	54.8	465,805	45.2
5–6 (5 min)	1022	537,507	56.7	310,618	43.3
6–7 (5 min)	1077	655,875	60.0	340,670	40.0

## Data Availability

All computational data is included in the manuscript. Videos links are provided in the Appendix A.

## Appendix A

- File link for “keratocyte.avi”, accessed on 11 July 2022:
  https://www.dropbox.com/s/pbcyos1voqjmx2x/keratocyte.avi?dl=0
  Caption for media file with file name “keratocyte.avi”:
  **Top-left**: the original image from experimental observation; **top-right**, our segmentation of the keratocyte from the image; **bottom-left**: we define the interfacial region of the cell and its centroid position, within this sub-figure, we continually overlay the cell shapes and positions as it migrates; **bottom-right**: we use colour coding to identify red as protrusion and blue as retraction forces and the locations where they are exerted on the cellular interfacial region, the bar on the right-hand side shows the maximum and minimum amount of forcing that the colour coding is illustrating. Bars indicate 20 μm.
- File link for “T24.avi”, accessed on 11 July 2022:
  https://www.dropbox.com/s/t0te420wx7niy0p/T24.avi?dl=0
  Caption for media file with file name “T24.avi”: **Top-left**: the original image from experimental observation; **top-right**, our segmentation of the T24 cancer cell from the image; **bottom-left**: we define the interfacial region of the cell and its centroid position, within this sub-figure, we continually overlay the cell shapes and positions as it migrates; **bottom-right**: we use colour coding to identify red as protrusion, and blue as retraction forces and the locations where they are exerted on the cellular interfacial region, the dark shadows in the background illustrate the targeted shapes that our model is trying to replicate, the bar on the right-hand side shows the maximum and minimum amount of forcing that the colour coding is illustrating. Bars indicate 20 μm.
- File link for “MDCK.avi”, accessed on 11 July 2022:
  https://www.dropbox.com/s/5gb7w1ddefczsm0/MDCK.avi?dl=0
  Caption for media file with file name “MDCK.avi”: **The first image on the first row**: the original image from experimental observation and our choice of three cells which are used in our simulation; **the second image on the first row**: our segmentation of the cells from the image on the left; **the first image on the second row**: we define the interfacial region of the cell and its centroid position, within this sub-figure, we continually overlay the cell shapes and positions as it migrates; **the second image on the second row**: we use colour coding to identify red as protrusion, and blue as retraction forces and the locations they are exerted on the cellular interfacial region, the dark shadows in the background illustrate the targeted shapes that our model is trying to replicate, the bar on the right-hand side shows the maximum and minimum amount of forcing that the colour coding is illustrating.; **the only image on the third row**: we show our Euler number from Equation (5) computed at each RTS and red circles indicate the events of cell division. Bars indicate 20 μm.
- Our approach to present simulation results The mathematical model in Equation (1) and its dimensionless form (i.e., Equation (4)) exist continuously in both space and time in the closed domain Ω. We apply discretisation schemes to obtain a discrete system at each reconstructed time step (RTS). For example, the situation presented in Figure 1 has been discretised into 10 time steps. Solving such a system yields its simulation result at its corresponding reconstructed time step. We use $\phi_{i}$ and $\eta_{i}$ to represent the discrete solutions on a grid point *i* where there are *N* grid points, and $\Omega_{L}$ is the number of grid points on one axis of the square domain Ω. $\phi_{i}$ describes the evolution of the cell shape and takes values either −1 or 1 in- or outside of the cell. When $\phi_{i}$ is between −1 and 1, it illustrates the phase-field interfacial region and the cell shape. The phase-field variable undergoes a smooth transition between the values of 1 and −1. The membrane corresponds to the zero level set of the phase field variable. In order to evaluate quantities on the membrane in our simulations results we need to specify the interfacial region from our simulation by considering a small interval around zero to be the interfacial region since our method does not explicitly track the interface itself. We define grid points *i* that has values of $\phi_{i}$ between −0.02 and 0.02 as the cell membrane. We evaluate the average length of the cell membrane on the interval *I*, where $I=\frac{t}{T_{I}}$ using the formula, $\left[\sum_{k=(I-1)*RTS+1}^{I*RTS}\sum_{i=1}^{N}\left\{\frac{L}{\Omega_{L}}|-0.02<\phi_{i}^{k}<0.02\right\}\right]/RTS$. $\eta_{i}$ is the forcing, and we are interested in these exerted closely around the cell membrane. The total protrusion force on the interval *I* is evaluated using the formula, $\sum_{k=(I-1)*RTS+1}^{I*RTS}\sum_{i=1}^{N}\left\{\eta_{i}^{k}|-0.02<\phi_{i}^{k}<0.02\;\;\mathrm{and}\;\;\eta_{i}^{k}>0\right\}$. The total retraction force on interval *I* is evaluated using the formula, $\sum_{k=(I-1)*RTS+1}^{I*RTS}\sum_{i=1}^{N}\left\{\eta_{i}^{k}|-0.02<\phi_{i}^{k}<0.02\;\;\mathrm{and}\;\;\eta_{i}^{k}<0\right\}$. The choice of RTS affects the stability of the solution methods, and we refer the reader to [34,35,36] for the computational details.
